# Hourly time series model in drowning-induced mortality in Guilan and Mazandaran provinces

**Published:** 2019-07

**Authors:** Enayatollah Homaie Rad, Hamid Heidari, Naeema Khodadadi Hassan-Kiadeh, Marieh Hosseinpour, Fatemeh Javadi, Leila Kouchakinezhad-Eramsadati

**Affiliations:** ^*a*^Guilan Road Trauma Research Center, Guilan University of Medical Sciences, Rasht, Iran.

## Abstract

**Background:**

Drowning is considered a major health problem in the world. According to World Health Organization statistics, drowning is one of the 10 main causes of death in the age range of 1-24 years in each region, and the third cause of death resulted from unintentional injuries in the world. The purpose of the study is to analyze drowning peak hours to help promote prevention policies.

**Methods:**

The present study used drowning data of Guilan and Mazandaran provinces in summer. The study model was estimated using a semi-panel non-linear Poisson regression model. The number of deaths was sorted according to the hours of drowning in three years 2014, 2015, and 2016. Finally, the predicted graphs of the hours of mortality and the regression model were estimated.

**Results:**

The regression model showed that the relationship between daytime hours and drowning mortality had a second-order pattern. The mortality increases during daytime hours, then it decreases. The IRR of time variable was 2.66 and significant, indicating a mean growth of 266% per pass of each hour of daytime. The hourly second-order IRR was 0.9689 and significant, indicating a decreasing hourly rate of drowning. The predicted peak point of drowning was at 15 o'clock.

**Conclusions:**

The study showed drowning peak time occurs at noon when it is warming and the swimmer may be neglected. Therefore, we can educate and made the society, especially parents, aware of the issue to help prevent and reduce the frequency of drowning.

**Keywords:**

Drowning, Time Series, Mortality

